# Cardiovascular Disease in HIV Patients: A Comprehensive Review of Current Knowledge and Clinical Implications

**DOI:** 10.3390/ijms26051837

**Published:** 2025-02-21

**Authors:** Sorina Șoldea, Maria Iovănescu, Mihaela Berceanu, Oana Mirea, Victor Raicea, Maria Cristina Beznă, Alexandru Rocșoreanu, Ionuț Donoiu

**Affiliations:** 1 Department of Cardiology, University of Medicine and Pharmacy Craiova, 200349 Craiova, Romania; sorina.iordache93@gmail.com (S.Ș.); maria.iovanescu25@gmail.com (M.I.); mihaela.florescu0222@yahoo.com (M.B.); oana.mirea83@gmail.com (O.M.); bezna.mariacristina@gmail.com (M.C.B.); alexandru.rocsoreanu@umfcv.ro (A.R.); ionut.donoiu@umfcv.ro (I.D.); 2 Department of Cardiovascular Surgery, University of Medicine and Pharmacy Craiova, 200349 Craiova, Romania

**Keywords:** HIV, cardiac disease, highly active antiretroviral therapies

## Abstract

Cardiovascular involvement in patients with human immune deficiency (HIV) has gained significant attention as the improved life expectancy of individuals with HIV has changed the paradigm regarding the long-term impact of the virus on cardiovascular health. We reviewed current literature on the prevalence, diagnosis, and unique characteristics of cardiovascular disease (CVD) in HIV patients, including those treated with protease inhibitors (PIs) and complementary therapies. The incidence of infectious, immunosuppressive, and nutritionally related pathologies in HIV patients has declined, largely due to advancements in highly active antiretroviral therapies (HAART) and supportive care. However, issues related to autoimmunity and chronic inflammation persist. Elevated levels of high-sensitivity C-reactive protein, along with activated cytokines and other pro-inflammatory molecules, are common in HIV patients and contribute significantly to the increased risk for endothelial dysfunction, coagulation disorders, and accelerated atherogenesis. The advent of HAART has significantly improved the prognosis for HIV patients, leading to prolonged life expectancy and a reduction in AIDS-related complications. However, this success has also resulted in a shift in the clinical presentation, with HIV patients showing more chronic and insidious cardiovascular manifestations.

## 1. Introduction

The clinical profile of patients with human immunodeficiency virus (HIV) infection has experienced significant changes within the past four decades. The severe opportunistic infections and the high prevalence of tumors such as Kaposi’s sarcoma were associated with premature death, with mortality rates up to 40%. The increase in disease awareness among subjects at risk, the improvement of early diagnosis, and the development of highly active antiretroviral therapy (HAART) contributed to a clear shift of demographics, primary causes of death, and comorbidities among subjects with HIV. Accordingly, it is estimated that by 2030, in the United States, 70% of the patients living with HIV will be older than 50 years of age [1]. Longer life expectancy will define a challenging group of patients that combine both classic age-related risk factors and HIV-related pathology, thus making cardiovascular disease one of the most prominent causes of death [1].

The present review includes contemporary knowledge of cardiovascular diseases in this category of patients, especially since the prevalence, type of cardiovascular disease, and pathophysiological mechanisms have suffered substantial variations in time. As such, this review will focus on myocardial and vascular diseases, as well as electrical disturbances reported in the evolution of HIV patients, using the currently existing literature data.

## 2. Myocardial Disease

The absence of a strict definition and the differences in availability of HAART between developed and developing countries make it difficult to estimate the incidence and prevalence of HIV-cardiomyopathy. This explains the varying results reported in the literature [2].

In the pre-HAART era, HIV-associated cardiomyopathy was primarily characterized by a dilated left ventricle (LV) with symptomatic systolic dysfunction (Central Figure). Most often, the underlying cause was repetitive myocarditis, which was related either to opportunistic infections, direct viral cytotoxicity, nutritional deficiencies, autoimmunity, or severe immune suppression [3].

One of the earliest studies on HIV-associated cardiomyopathy, conducted by Levy et al., found that 16% of 60 enrolled patients had systolic dysfunction, with 66% of them diagnosed with acquired immunodeficiency syndrome (AIDS) [4]. De Castro et al. evaluated the incidence of heart pathologies in HIV-infected patients during different stages of the disease. Of the patients who were in the end stage, 17% presented a dilated cardiomyopathy and 6% presented myocarditis, while 14% had regional cardiac abnormalities [5]. Similarly, Herskowitz et al. prospectively studied 69 patients without clinical heart disease, to find a 15% prevalence of global left-ventricular hypokinesia [6]. De Castro et al. identified acute left-ventricular dysfunction in 8% of AIDS patients without previous heart conditions. Of these, 85% died from congestive heart failure, and 57% were diagnosed with myocarditis through histology [7].

The introduction of HAART therapy increased life expectancy and reduced opportunistic infections. However, it also brought attention to other chronic HIV-related complications. As such, the presence of systolic dysfunction and especially the number of cases with severely reduced ejection fraction decreased, whereas diastolic dysfunction became more prevalent.

The Study to Understand the Natural History of HIV/AIDS in the Era of Effective Therapy (SUN Study) examined 656 asymptomatic HIV-infected participants who underwent echocardiography between 2004 and 2006. It found that 26% had diastolic dysfunction, compared to 18% with systolic dysfunction. Risk factors for diastolic dysfunction included high-sensitivity C-reactive protein (hsCRP) levels and hypertension, while myocardial infarction history, elevated hsCRP, and smoking were associated with systolic dysfunction [8].

A meta-analysis by Cerrato, which included 2242 HIV-positive patients with minimal symptoms, found a 44% incidence of diastolic dysfunction, with hypertension and older age as key predictors. Nearly all patients (98%) were on HAART, and cardiac imaging was performed at a median of 8.1 years post-diagnosis. The most common form of diastolic dysfunction was impaired relaxation (32%) [9].

## 3. Pathophysiologic Mechanisms

The multiple phenotypes of HIV-cardiomyopathy are associated with numerous pathophysiologic mechanisms, which seem to have changed over time according to the more nuanced types of myocardial involvement. The proposed causes include direct myocardial injury from HIV-1 (with or without myocarditis), opportunistic infections, nutritional deficiencies, autoimmune reactions, and drug toxicity [10].

One of the earliest theories linked HIV-1 infection directly to myocardial damage, particularly in relation to systolic dysfunction. Interestingly, some studies suggest that cardiomyocytes themselves are not the main target, but rather macrophages. This is likely because cardiac myocytes lack HIV-1 receptor proteins [11]. In a study by Twuet et al., researchers analyzed 18 hearts from AIDS patients (with and without cardiomyopathy) and found that those with cardiomyopathy had significantly higher levels of gp120 and tumor necrosis factor-alpha (TNF-α), both of which were associated with cardiomyocyte apoptosis. Both extrinsic and intrinsic apoptotic pathways were involved, as indicated by elevated caspase-9 fragments (linked to mitochondrial activation) and Fas ligand (a TNF superfamily member). Gp120 was highly expressed in macrophages and T cells but was either low or absent in cardiac myocytes. Additionally, experiments showed that while HIV-1 could enter rat cardiac myocytes via macropinocytosis in vitro, it was unable to replicate within them [12].

Beyond triggering cardiomyocyte apoptosis, TNF-α also impairs heart contractility by disrupting calcium-induced calcium release, as demonstrated by Yokoyama [13]. Furthermore, proinflammatory cytokines such as TNF-alfa trigger expression of nitric oxide synthase (iNOS), which has been recognized as being involved in cardiac myocyte depression [14,15]. This suggests that proinflammatory cytokines play a central role in the development of HIV-cardiomyopathy.

However, other cardiotropic viruses have the ability to invade cardiac myocytes, leading to myocarditis through localized cytokine release. The agents incriminated were frequently represented by Coxsackie B3 virus, Epstein-Barr virus, and cytomegalovirus [16]. Moreover, nonviral opportunistic agents were shown to be responsible for the development of myocarditis, especially in late stages of AIDS [3].

Autoimmune processes may further contribute to heart disease in HIV and AIDS patients. Specific cardiac autoantibodies, such as anti-alpha-myosin, have been detected in HIV-positive individuals. Viral infections, both opportunistic and common, might trigger autoimmunity by exposing cardiac surface epitopes. In a study by Currie et al., researchers compared 74 HIV-positive patients (both with and without structural heart disease) to HIV-negative controls. They found higher levels of anti-alpha-myosin autoantibodies in HIV-positive individuals, regardless of heart disease presence. However, within the HIV-positive group, those with structural heart disease had higher antibody concentrations (43%) compared to those without cardiac abnormalities (19%) [17].

Finally, while HAART has significantly improved HIV patient survival, it may also contribute to cardiac disease. Certain drug classes, such as protease inhibitors and some nucleoside reverse transcriptase inhibitors (NRTIs), have been associated with adverse cardiovascular effects, raising concerns about long-term heart health in HIV patients.

The use of zidovudine, a reverse nucleoside transcriptase inhibitor, was associated with focal myocardial necrosis, mainly through mitochondrial damage, but studies have failed to acknowledge the exact role of zidovudine in the pathogenesis of HIV-cardiomyopathy [18]. All four classes were associated with metabolic disturbances, such as lipodystrophy, hypertriglyceridemia, and insulin resistance. Nelson et al. found that HIV patients on HAART had three times the normal myocardial triglyceride content, which was linked to impaired cardiac function, as detected using advanced speckle tracking echocardiography [19].

## 4. Ischemic Heart Disease

### 4.1. Prevalence of IHD

The increase in life expectancy among HIV patients has led to a growing prevalence of both traditional cardiovascular risk factors—such as chronic tobacco use, hypertension, diabetes, and dyslipidemia—as well as HIV-specific factors, including chronic inflammation and immune suppression. This dual burden significantly raises cardiovascular disease risk in this population

Several studies have aimed to evaluate the risk of acute myocardial infarction (AMI) in HIV-positive individuals compared with HIV-negative controls.

A meta-analysis by Rao et al., which reviewed data from 16 longitudinal cohort studies involving HIV-infected adults, found that individuals with HIV have twice the risk of AMI compared to HIV-negative controls. This translates to an absolute increase of 2.2 cases per 1000 people per year [20]. Arterial hypertension, tobacco use, and dyslipidemia were the most important contributors to AMI risk.

Other meta-analyses and cohort studies have confirmed these findings. Moreover, several studies have identified an increased cardiovascular risk specifically associated with exposure to antiretroviral therapies [21,22,23,24].

However, some studies have reported different conclusions regarding myocardial infarction risk. A cohort study by Klein et al. [25], which analyzed data from 1996 to 2011, found that after adjusting for age, sex, diabetes, hypertension, and lipid-lowering therapy, there was no significant increase in AMI risk for HIV-positive individuals during the 2010–2011 period compared to matched HIV-negative controls.

This discrepancy may be explained by improvements in cardiovascular risk management, including a 30% increase in lipid-lowering and antihypertensive prescriptions. Additionally, better adherence to antiretroviral therapy and the achievement of very low viral loads (HIV RNA < 500 copies/mL) may have reduced overall inflammation, thereby lowering cardiovascular risk [25].

The potential differences in AMI outcomes—both in-hospital mortality and 1-year prognosis—have drawn significant attention. Lorgis et al. [26], who examined AMI patients hospitalized between 2005 and 2009, found significantly lower hospital and 1-year mortality rates in the HIV-infected group compared to HIV-negative individuals (3.1% vs. 8.1%, *p* < 0.001 for hospital mortality; 1.4% vs. 5.5%, *p* < 0.001 for 1-year mortality). However, ischemic cardiomyopathy and heart failure hospitalizations at 12 months were more common in the HIV group (3.3% vs. 1.4%).

There was a male sex predominance (88% of the HIV patients), and the most prevalent risk factors were smoking and dyslipidemia. Interestingly, HIV-positive individuals had a lower prevalence of diabetes and hypertension compared to HIV-negative individuals, a trend also observed in studies by Boccara et al. and Hsue et al. [27,28]. These findings suggest that while the short-term risk of AMI is similar between HIV-positive and HIV-negative individuals, HIV status may influence long-term cardiovascular outcomes [26].

### 4.2. Mechanisms of IHD

Atherosclerosis remains the main cause of ischemic heart disease (IHD). It is well-known that atherosclerosis is a chronic inflammatory, active process characterized by the development of arterial atherosclerotic plaques. A key contributor to this condition is hypercholesterolemia, where high levels of low-density lipoprotein (LDL) cholesterol initiate endothelial dysfunction, promote lipid accumulation, and lead to the buildup of macrophage foam cells in the subendothelial space.

Research has shown a negative association between antiretroviral therapy and early-onset atherosclerosis [29]. Seminari and colleagues found that HIV patients receiving protease inhibitors had higher triglyceride and apoB levels. Additionally, these patients exhibited significantly increased carotid intima-media thickening (IMT) compared to untreated or HIV-negative individuals [29].

The introduction of HAART led to an increased incidence of lipodystrophy syndrome, a condition characterized by hyperlipidemia and impaired glucose tolerance—both well-known risk factors for coronary artery disease in both HIV-positive and HIV-negative individual [30,31,32].

Table 1 lists the most common adverse effects of HIV medications.

However, these findings were not confirmed in a systematic review and meta-analysis of observational studies conducted by Hulten et al. Their analysis of 26 studies focused on subclinical atherosclerotic lesions assessed via carotid ultrasound or coronary artery calcium scores in patients receiving protease inhibitors versus non-recipients. They concluded that PI exposure did not significantly impact carotid intima-media thickness (IMT) or coronary calcium scores [42]. However, for patients interrupting treatment, there was a 70% increased hazard of cardiovascular events [43].

A separate large prospective study spanning seven years investigated the link between HIV infection and the progression of subclinical atherosclerosis. After adjusting for demographic, behavioral, and cardiometabolic risk factors, the study found that HIV-infected individuals had a 1.6-fold greater risk of developing new plaques compared to uninfected individuals. Notably, this increased risk was observed even in those with optimal viral suppression, suggesting the involvement of additional complex mechanisms [44].

Another study found that young, asymptomatic men with long-standing HIV infection had a twofold higher prevalence and burden of coronary atherosclerosis (24% vs. 12%) compared to non-infected individuals, as assessed by coronary computed tomography [45].

Moreover, a higher prevalence of non-calcific coronary plaques in HIV-positive patients with low CD4+ T-cell counts was reported, suggesting the role of systemic inflammation and immune activation [46]. Fluorodeoxyglucose positron emission tomography (18F-FDG-PET) imaging further supported these findings by revealing increased arterial inflammation in HIV-positive individuals. Additionally, plasma-soluble CD163 (sCD163), a monocyte/macrophage-specific activation marker, correlated with 18F-FDG-PET data, indicating a higher presence of inflammatory macrophages in the ascending aortas of HIV-infected individuals [47].

Kearns and colleagues proposed potential mechanisms for HIV-associated atherosclerosis, emphasizing the role of endothelial dysfunction as the initial step. Endothelial dysfunction promotes the release of prothrombotic and proinflammatory cytokines (IL-6, TNF) and adhesion molecules (VCAM-1, P-selectin), which amplify inflammation and attract monocytes (pro-atherogenic CD-14+, CD-16+) and T cells. Once inside the intima, macrophages engulf oxidized low-density lipoproteins (oxLDL), forming foam cells and leading to plaque development—the hallmark of atherosclerosis. Since they remain chronically infected, monocytes and macrophages seem to be some of the leading cellular players in HIV-atherosclerosis, where the virus, although in a latent state due to HAART, can affect their function through potent viral regulatory proteins [48,49,50,51]. Regarding molecular mechanisms, several factors, such as increased oxidative stress, inflammasome formation and dysregulation of autophagy, are included, but they are beyond the scope of this review.

## 5. Pericardial Disease

Before the introduction of HAART, pericarditis was diagnosed in approximately 11% of HIV patients, making it one of the most common cardiovascular complications in this population. In patients with AIDS, the prevalence was even higher [52].

Pericardial effusion can have multiple causes. An older study identified the etiology in 24% of cases, with the most common causes being Coccidioides, Mycobacterium tuberculosis, or tumors [53]. Another study focusing on patients with extrapulmonary tuberculosis found pericarditis in only 5% of cases, suggesting it is a relatively rare complication [54].

In AIDS, the risk of opportunistic infections is significantly increased. Case reports have documented pericarditis caused by atypical mycobacteria, fungi, viruses (such as cytomegalovirus and herpes simplex), and protozoa (Toxoplasma gondii). These infections are often difficult to diagnose and treat. In AIDS patients, the presence of pericardial effusion—rather than its volume—has been associated with a poor prognosis [52].

## 6. Infective Endocarditis

Most cases of infective endocarditis (IE) in HIV patients occur in young individuals with a history of intravenous drug use. In an analysis by Cicalini, *Staphylococci* were responsible for over 60% of cases, with the infection primarily affecting the tricuspid valve in 52% of patients [55].

In the absence of IV drug use, infection affects mainly the mitral and aortic valves, and in more than half of cases, it occurs in advanced disease. A diversity of microorganisms was associated with IE, including *Enterococcus fecalis*, *Streptococcus viridans*, coagulase-negative *Staphylococci*, *Staphylococcus aureus* (found on infected catheters or skin and soft tissue infections), *Salmonella* sp., *Streptococcus pneumoniae* (common causes of bacteremia in HIV patients), and fungi [56].

The mortality rate of HIV patients with IE is comparable to that of the general population with infective endocarditis. However, HIV-positive patients are less frequently referred for surgical intervention [56].

## 7. Pulmonary Hypertension

Pulmonary arterial hypertension (PAH) is a rare but serious HIV complication, affecting ~0.5% of patients [57,58]. It causes vascular remodeling similar to idiopathic PAH, including endothelial and smooth muscle hyperplasia, intimal fibrosis, thrombi, and plexiform lesions. Inflammatory infiltrates (lymphocytes, histiocytes) are common, while alveolar septal thickening and interstitial fibrosis may also occur [57].

Various imaging techniques (electron microscopy, immunohistochemistry, in situ hybridization, PCR) have not detected HIV in vascular wall cells. However, viral proteins are present in lung tissue and likely contribute to PAH development [57].

The viral proteins incriminated are glycoprotein 120 (gp 120), trans-activator of transcription (Tat) and negative factor (Nef) proteins, each having an important role in infection.

Gp 120 is found in viral membranes and mediates the attachment to the host cell. It stimulates cytokine release from macrophages, increases endothelin-1 synthesis, and triggers endothelial cell apoptosis [59].

Trans-activator of transcription protein causes injury to the endothelial cells. Tat attaches to the endothelial cells through integrins and the type 2 receptor for VEGF. Tat and gp120 stimulate the production of hypoxia-inducible factor 1-alpha (HIF-1α) and platelet-derived growth factor-β (PDGF-β), both of which promote cell proliferation.

Nef protein is synthesized early in viral infection and mediates disease progression. It downregulates major histocompatibility complex 1 (MHC-I) and CD4 receptors, weakening the immune response. Instead of recycling, CD4 receptors are broken down inside lysosomes. Nef also inhibits cell mobility, stimulates CD4+ T-cell apoptosis, and has been linked to the structural changes observed in PAH. Specific Nef mutations have been identified in HIV patients who develop PAH [59].

Other factors incriminated in PAH pathogeny are drugs (heroin, morphine, cocaine) that cause injury of the endothelial cells and co-infections with microorganisms like *Mycobacterium tuberculosis*, *Pneumocystis carinii*, *Schistosoma mansoni*, which all cause lesions of the lung parenchyma and lead to persistent lung inflammation [57].

In Sitbon et al.’s study, PAH prevalence in HIV patients was 0.46% (35/7648 cases). Mean pulmonary artery pressure (PAP) was 46 ± 13 mmHg in existing PAH and 30 ± 9 mmHg in new cases. Better outcomes were seen in patients with CD4 > 200 cells/mL and cardiac index ≥ 2.8 L/min/m^2^ [60].

Recent advancements in the treatment of pulmonary arterial hypertension (PAH) in individuals with HIV have focused on integrating novel therapeutic agents and optimizing existing treatment strategies to improve patient outcomes. HIV patients do not respond to the vasodilating test, so calcium channel blockers are not useful. Traditional PAH treatments, such as endothelin receptor antagonists (e.g., bosentan, ambrisentan) and phosphodiesterase-5 inhibitors (e.g., sildenafil, tadalafil), continue to be integral in managing HIV-associated PAH. These therapies have demonstrated efficacy in improving hemodynamics and exercise capacity. However, clinicians must remain vigilant regarding potential drug–drug interactions between PAH-specific medications and antiretroviral therapies, as well as the unique side effect profiles in HIV-infected individuals. Anticoagulation therapy is generally avoided because of the increased risk of bleeding and interactions with antiretroviral medications [60]. Ongoing research is essential to further understand the pathophysiology of HIV-associated PAH and to develop targeted therapies. Future studies should focus on the long-term efficacy and safety of emerging treatments like sotatercept in the HIV-positive population, as well as the potential benefits of combination therapies.

## 8. Thromboembolic Disease

Thromboembolic disease in HIV patients varies widely (0.19–7.63%), with a 2–10 times higher risk than the general population [61]. In a study by Malek, HIV patients had a 43% increased risk of pulmonary embolism and a 10% higher risk of deep vein thrombosis (DVT) than age-matched HIV-negative individuals. The highest risk was observed in patients aged 21 to 50 years [62].

HIV infection contributes to a prothrombotic state and endothelial dysfunction. The prothrombotic tendency is mainly due to anticoagulant factor deficiencies, particularly Protein S deficiency. Its prevalence in HIV patients ranges from 2% to 76%, significantly higher than in the general population. However, only 12% of affected patients develop thromboembolism.

Type III Protein S deficiency is most frequently observed, characterized by normal total Protein S levels but reduced free Protein S levels and decreased functional activity. This deficiency results from reduced hepatic, endothelial, and megakaryocyte synthesis due to HIV-induced damage. Elevated TNF-α further inhibits Protein S production, and autoantibodies against Protein S have also been detected in HIV patients [61].

HIV infection can trigger low-grade disseminated intravascular coagulation due to prolonged immune stimulation and injury of the endothelial cells, which leads to consumption of the clotting factors and anticoagulant factors.

Deficiencies of antithrombin III and Protein C are less common and are usually linked to preexisting liver disease (e.g., co-infections with Hepatitis B or C) or AIDS-related malnutrition and protein loss (due to enteropathies or nephropathies) [61].

Additionally, prolonged immune activation can trigger antiphospholipid antibody production. Lupus anticoagulant and anticardiolipin antibodies are detected in 7% to 94% of HIV patients [61].

HIV infection, along with proinflammatory cytokines, contributes to endothelial dysfunction. In response, endothelial cells increase the expression of adhesion molecules (P-selectin, E-selectin) and enhance the release of procoagulant and pro-aggregant factors (von Willebrand factor, thrombomodulin, tPA, PAI-1, fibronectin) as well as vasoconstrictors (angiotensin II, endothelin-1). Additionally, tissue factor expression on monocytes is elevated due to excessive immune stimulation, further promoting arterial and venous thrombosis [61].

The risk of thromboembolism correlates with HIV disease severity. Patients with low CD4+ counts and high viral RNA loads face the greatest risk. In AIDS-stage HIV, various opportunistic infections (cytomegalovirus, *Pneumocystis jirovecii*, *Mycobacterium avium-intracellulare*) further increase the thrombotic risk [61].

HAART has substantially improved the prognosis of HIV patients. Older case reports described the occurrence of thromboembolism shortly after the initiation of therapy with protease inhibitors, in particular indinavir, raising concerns that PIs might induce endothelial dysfunction and create a procoagulant status. However, more recent analysis did not find any correlation between thrombosis occurrence and antiviral therapy, nor between its duration or the duration of HIV infection [63].

In a study by Crum-Cinaflone, 3.7% of HIV patients experienced an embolic event, with 23% of these being pulmonary embolisms. Almost all affected patients had classical thrombotic risk factors, with 59% having concurrent infections. Additionally, patients with thrombosis had lower CD4+ counts and higher viral loads and were more likely to have AIDS than those without thromboembolism [64].

The role of biomarkers in diagnosing PE among HIV patients is an area of ongoing research. Elevated D-dimer levels are commonly used to assess PE risk, but HIV infection itself can influence D-dimer concentrations due to chronic inflammation and immune activation. Further studies are needed to determine appropriate D-dimer thresholds and to explore additional biomarkers that may improve diagnostic specificity for PE in HIV-positive individuals.

Thromboembolic therapy is similar for patients with and without HIV, but the clinician should be aware of some drug interactions. Heparins are safe to use. Non-nucleoside reverse transcriptase inhibitors (NNRTI) and some PIs act on CYP2C9 enzyme, that is responsible for the metabolism of warfarin (some compounds may increase or reduce the warfarin concentration), causing large fluctuations of the anticoagulant effect. Patients undergoing warfarin therapy need frequent INR monitoring [61].

NOACS also interact with PIs. Factor X inhibitors (apixaban, rivaroxaban) are transported through the membrane by glycoprotein P (gpP) and metabolized by CYP 3A4. Some PIs (e.g., ritonavir) inhibits CYP3A4 and gpP and can significantly increase serum concentration of NOACS and also the bleeding risk. The co-administration of PIs and factor X inhibitors is contraindicated. Dabigatran, a factor IIa inhibitor, uses gpP for membrane transport and should not be used together with PIs.

## 9. Cardiac Tumors

HIV-infected individuals have a higher risk of developing malignancy through different pathogenic pathways: immunosuppression, direct effects of the virus, coinfection with other oncogenic viruses (Ebstein-Barr virus, Hepatitis B and C viruses, human papillomavirus, etc.), or increased sensitivity to environmental factors like tobacco. There is also a possible link between HAART and some cancers [65]. Cardiac neoplasms are generally a manifestation of metastatic disease. Heart malignancy has been found in 28% of HIV-infected patients, most necropsy cases being Kaposi’s sarcomas, and is relatively rarely described as a primary heart tumor. The primary involvement of the heart associated with HIV is mainly due to lymphomas—in 1990, non-Hodgkin lymphoma (NHL) became an HIV-defining condition. Non-Hodgkin lymphomas are estimated to have a 25–60-fold higher incidence in HIV-infected people and are the first manifestation of the disease in over 3–4% of new cases, with a marked decline in the era of HAART. Multiple risk factors have been identified in the development of non-Hodgkin lymphoma.

In 1983, Autran described the first case of cardiac Kaposi’s sarcoma in a patient with HIV infection [66]. On retrospective necropsy, the incidence of cardiac Kaposi’s sarcoma was 12–28% [67]. The disease affects the visceral sheet of the pericardial serosa or the subepicardial adipose tissue. There is a predilection for Kaposi’s sarcoma to affect the subepicardial fat adjacent to a major coronary artery, with or without the involvement of the ascending aorta or pulmonary artery trunk.

In 1985, the US Centers for Disease Control and Prevention recognized the link between intermediate and high-grade lymphomas and HIV-positive people and included them in the diagnostic criteria for AIDS.

Malignant lymphomas are not very common in AIDS, but they are the second most frequent neoplasm affecting the heart [68]. Lymphoma infiltration may be diffuse or may be organized as discrete isolated lesions, usually derived from Burkitt cells or B-type immunoblastic cells. Cardiac lymphoma—the disseminated form—is more common than the primary cardiac form. It is estimated to be responsible for 15% of all cardiac and pericardial metastases in HIV-infected patients. The HIV immune impairment is considered to be the primary mechanism, with patients having high HIV-RNA viral loads, together with CD4 of less than 110 cells/μL [69]. Primitive cardiac lymphoma is extremely rare; patients usually have nonspecific symptoms, but after their onset, heart failure progresses rapidly. At the time of detection, patients may present with refractory heart failure, pericarditis, cardiac tamponade, or cardiac arrhythmias [70,71].

The most common tumor presentation is in the form of polypoid or nodular masses that mainly affect the pericardium, with varying degrees of myocardial infiltration [72]. From a histological point of view, they are diffuse, aggressive forms of lymphoma, generally with small, undifferentiated, or immunoblastic cells [73,74]. Patients with mechanical obstructive forms may benefit from surgical resection. The prognosis of patients with HIV-associated cardiac lymphoma is usually poor, although cases of clinical remission have also been reported after combined chemotherapy. Even though the heart can be considered a primary lymphoma site, in many HIV patients, the cardiac involvement usually represents a metastatic process.

## 10. HIV and Atrial Fibrillation

Early studies have established a strong link between HIV infection severity and the risk of developing atrial fibrillation (AF). Studies suggest that inflammatory cytokines contribute to atrial remodeling and electrophysiological changes, increasing the likelihood of AF development. Echocardiography and cardiac MRI derived data have demonstrated that HIV-infected individuals often exhibit left atrial enlargement and fibrosis, which creates an environment favoring the abnormal electrical conduction and AF development.

Interestingly, some studies suggest that traditional stroke risk scores, such as CHA_2_DS_2_-VASc, may underestimate the thromboembolic risk in HIV patients, indicating the need for HIV-specific risk stratification models [75]. Moreover, AF seems to have a higher recurrence in HIV patients after catheter ablation compared to controls, requiring a personalized treatment approach [76].

## 11. Future Research and Clinical Practice in HIV

As HIV care continues to evolve, further research to optimize cardiovascular and thromboembolic disease management in HIV-positive individuals is needed. Key areas are the development of HIV-specific cardiovascular risk scores that could incorporate both traditional and HIV-related risk factors. Also needed are long-term prospective studies to evaluate the cardiovascular effects of newer antiretroviral drugs, particularly integrase inhibitors and next-generation protease inhibitors, as well as the creation of individualized treatment regiments that minimize cardiovascular risks while effectively managing HIV.

Furthermore, studies focused on long-term safety and efficacy of NOACs in HIV patients and identify safer anticoagulation strategies with fewer drug interactions are warranted.

## 12. Conclusions

HIV infection and its treatment significantly influence cardiovascular health. A multidisciplinary approach, incorporating both HIV management and cardiovascular prevention, is essential to improve outcomes and reduce the burden of cardiovascular disease in this population.

## Figures and Tables

**Table 1:** Common cardiovascular adverse effects of HIV medications.

HIV Medication Class	Drug Examples	Adverse Cardiovascular Effects
Nucleoside Reverse Transcriptase Inhibitors (NRTIs) [33,34,35]	abacavir, didanosine, lamivudine, emtricitabine, zidovudine, stavudine	Higher risk for myocardial infarct controverted in studies Dyslipidemia Insulin resistance
Non-nucleoside reverse transcriptase inhibitors (NNRTIs) [36,37]	efavirenz, nevirapine, etravirine, rilpivirine, doravirine	Higher risk for myocardial infarct Accelerated atherosclerosis Rare QT prolongation. Hypercholesterolemia hypertriglyceridemia
Protease inhibitors (PIs) [38,39]	atazanavir, darunavir, lopinavir/ritonavir, ritonavir, saquinavir, tipranavir	Increased mortality in subjects with HF Hyperglycemia Hypercholesterolemia Hypertriglyceridemia Accelerated atheromatosis
Integrase strand transfer inhibitors (INSTIs) [40]	dolutegravir, bictegravir, raltegravir, elvitegravir	QT prolongation Hypercholesterolemia Weight gain Arterial hypertension
Entry inhibitors [41]	maraviroc, enfuvirtide, ibalizumab, fostemsavir	Hypotension Rare QT prolongation
